# High‐Throughput Sequencings Revealed That Gut Microbiota Dysbiosis is Implicated in Gouty Arthritis of Red‐Crowned Crane (*Grus japonensis*)

**DOI:** 10.1155/tbed/2422900

**Published:** 2025-12-15

**Authors:** Hong Lin, Xiaoyang Zhu, Jianzhong Zhu, Nanhua Chen, Wenbin Bao

**Affiliations:** ^1^ Key Laboratory for Animal Genetics, Breeding, Reproduction and Molecular Design, Department of Animal Husbandry, College of Animal Science and Technology, Yangzhou University, Yangzhou, 225009, China, yzu.edu.cn; ^2^ Department of Preventive Veterinary Medicine, College of Veterinary Medicine, Yangzhou University, Yangzhou, 225009, China, yzu.edu.cn; ^3^ Joint International Research Laboratory of Agriculture and Agri-Product Safety, Yangzhou University, Yangzhou, 225009, China, yzu.edu.cn; ^4^ Jiangsu Co-Innovation Center for Prevention and Control of Important Animal Infectious Diseases and Zoonoses, Yangzhou University, Yangzhou, 225009, China, yzu.edu.cn

**Keywords:** 16S rRNA gene sequencing, gouty arthritis (GA), gut microbiota dysbiosis, gut-joint axis, MALDI-TOF/MS, metagenomics sequencing, red-crowned crane, treatment

## Abstract

The red‐crowned crane (*Grus japonensis*) is one of the rarest cranes with a global population of less than 4000 individuals. The population of red‐crowned crane could be influenced by health threats, including metabolic and infectious diseases. In the Wildlife Rescue Center of Suining County of Jiangsu Province, gouty arthritis (GA) was observed in all four red‐crowned cranes since March 2024. A pooled fecal supernatant was first submitted to metagenomics sequencing for screening disease‐associated pathogens. Enterobacteria phage phiEcoM‐GJ1 was detected as the predominant virus while *Escherichia coli* and *Aeromonas hydrophila* were the dominated bacteria in the mixed fecal sample from red‐crowned cranes. The 16S rRNA gene sequencing was further performed on both the mixed fecal sample and four individual samples, which showed that *Escherichia-Shigella*, *Lactobacillus*, and *Enterococcus* were the most abundant gut flora in both mixed and individual fecal samples. Furthermore, bacteria isolation and identification with matrix‐assisted laser desorption ionization‐time of flight mass spectrometry (MALDI‐TOF/MS) confirmed that *Escherichia coli* was predominant (19/29 colonies, 65.52%) in the feces. Therefore, anti‐uricacid and antibacteria treatments using plantain herb, doxycycline, Vitamin AD3 and multivitamin B were adopted, leading to a full behavioral recovery within 1 month. Overall, this case‐based observational study provides first clue on the gut‐joint axis in red‐crowned cranes, supporting that gut microbiota dysbiosis is closely associated with GA in red‐crowned cranes.

## 1. Introduction

The red‐crowned crane (*Grus japonensis*) is a large, wading, omnivorous bird in the family of Gruidae, which serves as an indicator species of the wetland environment [1]. In 2021, the red‐crowned crane has been designated as vulnerable (VU) in the International Union for Conservation of Nature (IUCN) Red List. Moreover, it’s also listed in the National Class I Key Wildlife Protection of China’s National List of Key Wildlife Protection [2]. The International Crane Foundation (ICF) estimates that the current population in the wild globally is about 2800–3430 individuals [3]. The number of red‐crowned cranes is gradually decreasing mainly due to the massive loss and deterioration of habitat and health threats, including metabolic and infectious diseases [4].

Gouty arthritis (GA) is a complex disorder caused by the monosodium urate (MSU) crystal formation and deposition intra and periarticularly [5]. GA is an important public health problem impacting human beings who suffer from dramatic joint inflammation and excruciating arthritic pain [6]. Similarly, GA also influences the health condition of animals, such as chicken and mouse [7, 8]. Remarkably, poultry (a kind of bird such as chicken) and human not only have similar purine nucleotide metabolic pathways, but also have some commons in uric acid synthesis and metabolism [8]. Therefore, poultry can serve as an ideal model to study GA. Moreover, GA treatment in animals may also provide a reference for effective treatment of GA in human. However, GA in red‐crowned crane (an omnivorous bird) has not been described yet.

The gut microbiome participates in diverse physiological processes, such as nutrient metabolism, immune modulation, host defense, and intestinal barrier integrity maintaince [9, 10]. Upon gut microbiota dysbiosis, detrimental taxa proliferate excessively and elaborated metabolites may trigger GA onset [11]. In addition, gut microorganism and metabolite alterations can impact GA therapeutic efficacy [9]. Intriguingly, a latest study confirmed the existence of a functional gut‐joint axis [12]. So far, the influence of intestinal microorganisms on GA in red‐crowned crane is also unknown.

In this study, we evaluated the precipitating factors for GA in four red‐crowned cranes from the Wildlife Rescue Center of Suining County of Jiangsu Province. Metagenomics sequencing was first used to screen GA‐associated pathogens in a pooled fecal sample, which were further confirmed by 16S rRNA gene sequencing. Moreover, bacteria in the red‐crowned crane feces were cultured and identified by matrix‐assisted laser desorption ionization‐time of flight mass spectrometry (MALDI‐TOF/MS). Finally, anti‐uricacid and antibacteria treatments were adopted for these red‐crowned cranes. Overall, our findings supported that GA in these red‐crowned cranes is closely associated with gut microbiota dysbiosis.

## 2. Materials and Methods

### 2.1. Ethics Statement

Fecal samples were collected via a noninvasive method to avoid unnecessary human interference with red‐crowned cranes, which was authorized by the Wildlife Rescue Center of Suining County of Jiangsu Province.

### 2.2. Sample Collection and Pretreatment

Since March 2024, all four red‐crowned cranes in the Wildlife Rescue Center of Suining County of Jiangsu Province showed clinical signs of limping and depressing. The joint abnormalities were captured in an X‐ray of feets using YEMA Clear X vet DR50 (Huanxi Medical Equipment, Shanghai, China). The blood biochemical indices were determined by VetScan VS2 Chemistry Analyzer (Abaxis, Union city, CA, USA). In addition, four fresh fecal samples were collected immediately after defecation from each red‐crowned cranes in May 10, 2024. Feces were stored in the 1.5 mL sterile centrifuge tubes and quickly brought back to our laboratory in Yangzhou University. Each fecal sample was resuspended in 1 mL of phosphate buffered saline (PBS) and vortexed for 5 min. After centrifugation at 10,000 × rpm for 10 min, each supernatant was collected for storage in −80°C until use.

### 2.3. Metagenomics Sequencing

Metagenomics sequencing was utilized to screen for potential GA‐associated pathogens using both DNA and RNA extracted from a pooled fecal sample at Tanpu Biotechnology Co. Ltd (Shanghai, China) [13]. In details, equal volume of fecal supernatants from four red‐crowned cranes were mixed to prepare a mixed sample. DNA and RNA were extracted from the mixed fecal sample using HiPure Tissue DNA Mini Kit (Magen, Guangzhou, China) and TRIpure reagent (Aidlab, Beijing, China), respectively. RNA was firstly fragmented via Mg^2+^ and then reverse transcribed into double stranded cDNA (ds cDNA) using the PrimeScript first Strand cDNA Synthesis Kit (TaKaRa, Japan). DNA was fragmented with Covaris M220. Both fragmented cDNA and DNA were submitted to library preparation using TruSeq DNA Sample Prep Kit (Illumina, USA). Bridge PCR was performed using TruSeq PE Cluster Kit (Illumina, USA). Illumina Novaseq 6000 PE150 sequencing was executed to generate raw data. Clean reads was generated by removing low‐quality reads, reads containing adapters and reads containing poly‐N from the raw data. Moreover, host contamination, the ribosome RNA and bacteria sequences were also filtered by using bbmap. The de novo assembly and assembly evaluation was performed using SPAdes and MEGAHIT. The extracted assembled scaffolds were subjected to sequence annotation in NCBI nt database.

### 2.4. Real‐Time PCR Detection

The dominant bacteria detected by metagenomics sequencing were double checked by real‐time PCR using primers shown in Supporting Information Table S1 [14]. Briefly, DNA was extracted from the mixed fecal sample using HiPure Tissue DNA Mini Kit (Magen, Guangzhou, China). Bacteria real‐time PCR detection was performed using the Premix Ex Taq (TaKaRa, Japan) and StepOne Plus Real‐Time PCR System (Applied Biosystems, USA) in according with the manufacturer’s instructions.

### 2.5. 16S rRNA Gene Sequencing

To further confirm the bacteria results generated from metagenomics sequencing, 16S rRNA gene sequencing was also performed on one mixed and four individual fecal supernatants from red‐crowned cranes as previously described [15, 16]. In details, bacterial DNA was extracted with HiPure Tissue DNA Mini Kit (Magen, Guangzhou, China). The DNA concentration was evaluated with Nano‐400A micro‐spectrophotometer (Allsheng, Hangzhou, China) and the DNA quality was double checked by 1% agarose gel electrophoresis. The 16S rRNA gene V3‐V4 variable region was PCR amplified with TransStart FastPfu DNA polymerase (TransGen, Beijing, China) using the 341F/806R primer pair (341F: 5’‐CCTAYGGGRBGCASCAG‐3’, 806R: 5’‐GGACTACNNGGGTATCTAAT‐3’). The amplicons were purified with Agencourt AMPure XP beads (Beckman Coulter, Brea, USA), quantified with the Quantus Fluorometer (Promega, Madison, USA), and then used for library preparation. The obtained libraries were pooled and submitted to sequencing via the Illumina MiSeq PE300 platform (SanDiego, USA) according to the manufacturer’s protocols at Tripanicum Technology Co. Ltd (Shanghai, China).

### 2.6. Identification by MALDI‐TOF/MS

The bacteria in the pooled fecal sample were isolated and identified by MALDI‐TOF/MS using the MALDI‐TOF Microflex LT Biotyper (Bruker, Bremen, Germany) as recently described [17]. In details, the mixed fecal supernatant of red‐crowned cranes was inoculated onto three commercial culture plates (blood agar plate (Huankai Microbial Co. Ltd, Guangdong, China), Macconkey agar plate (Huankai Microbial Co. Ltd, Guangdong, China), and nutrient agar plate (Bio‐way Technology Co. Ltd, Shanghai, China), and incubated for ~24 h at 37°C. Single colony from each fresh cultured plate was spread at designated locations on a standard MALDI target plate. After adding 1.0 μL 70% formic acid, the plate was left to air dry for ~5 min at room temperature. A volume of 1.0 μL HCCA mMatrix (saturated solution of *α*‐cyano‐4‐hydroxycinnamic acid in 50% acetonitrile, 2.5% trifluoroacetic acid) was added on the top of each spot within 10 min after drying. And then, the target plate was inserted into the Bruker Microflex LT MALDI‐TOF/MS system for analysis. A composite profile of proteins with m/z of 3000 to 15,000 was generated based on a minimum of 240 measurements (laser shots) for each specimen. The composite profile was analyzed using Biotyper 3.0 software (Bruker, Bremen, Germany), which queried a reference bank of 3995 spectra and returned the top 10 identification matches along with confidence scores ranging from 0.0 to 3.0. Scores ≥ 2.0 were considered high‐confidence (secure species) identification, scores of 1.7–1.99 were considered intermediate‐confidence (genus only) identification, and scores < 1.7 were considered unacceptable identification.

### 2.7. Anti‐Uricacid and Antibacteria Treatments

Based on clinical symptoms and detection results, the following therapeutic regimen was provided by the Animal hospital at Yangzhou University. The diseased red‐crowned cranes were treated with plantain herb (0.6–1 g/kg of body weight), doxycycline (15–20 mg/kg of body weight), Vitamin AD3 (300,000–500,000 IU/Kg of feed) and multivitamin B (100 g/ton of feed) for 1 month. Retrospective inquiry was adopted after 1 month treatment.

### 2.8. Statistical Analysis

The data were presented as mean ± SD. Intergroup comparisons were calculated using one‐way ANOVA in GraphPad Prism v8.0.3 [18].

## 3. Results

### 3.1. Clinical Investigations of the Diseased Red‐Crowned Cranes

In the Wildlife Rescue Center, we observed that red‐crowned cranes trudged in the plain road (Figure 1A). X‐ray detection showed that joint swelling and wear and tear in the left feet (Figure 1B). Noticeably, the comprehensive clinical chemistry results showed that the uric acid in blood reachs a extremely high level of 17.3 mg/dL (~1029 μmol/L, the normal level generally ranges from 100 μmol/L to ~400 μmol/L) (Figure 1C). These results led to a clinical diagnosis of GA in these red‐crowned cranes.

Figure 1Clinical investigations of GA in red‐crowned cranes. (A) Clinical observation identified limping in a representative red‐crowned crane. (B) X‐ray detection of feets using YEMA Clear X vet DR50 found joint swelling and wear and tear in the left feet. (C) The detection of blood biochemical indexs by VetScan VS2 Chemistry Analyzer confirmed the hyperuricemia.

**Figure:**
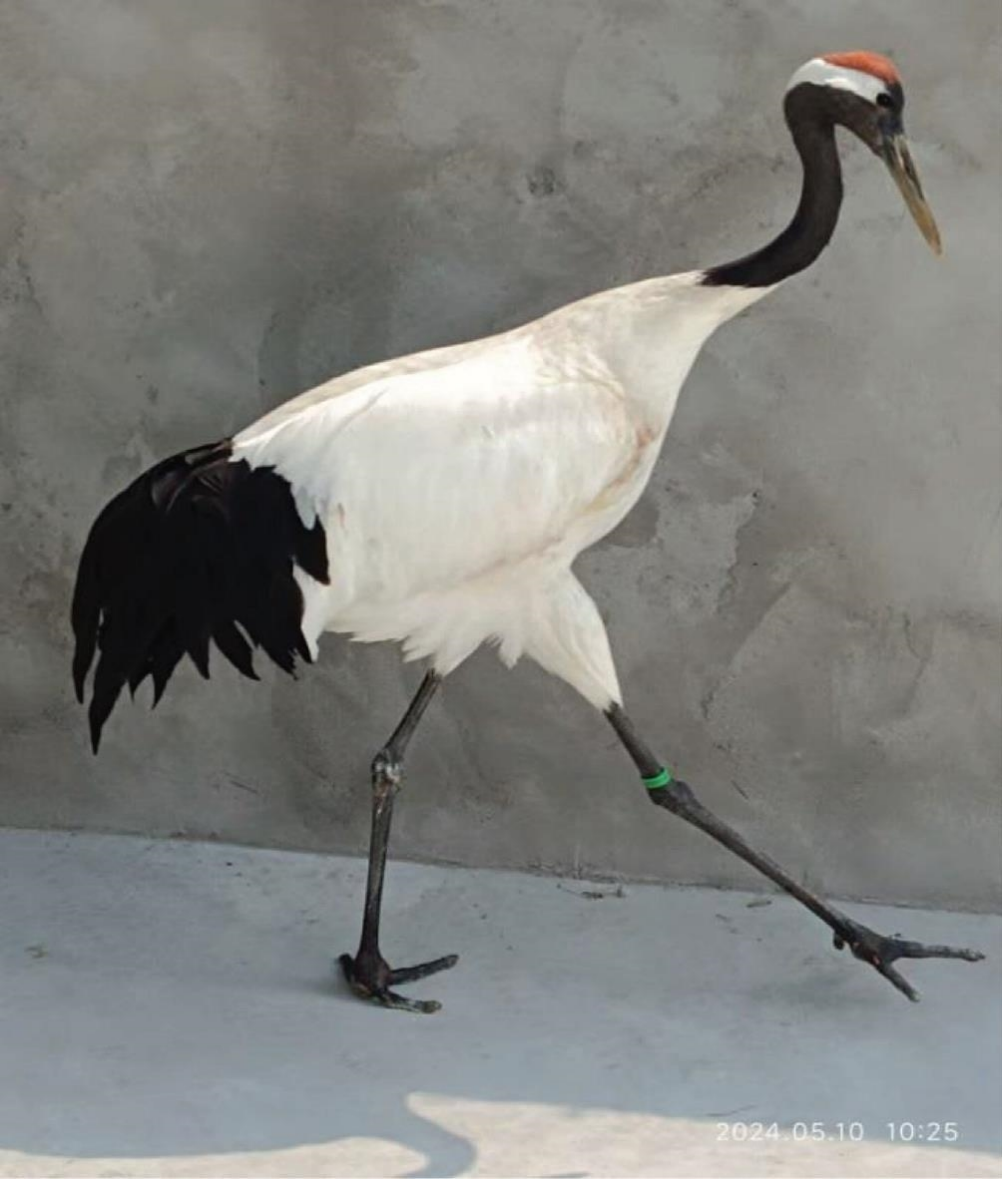
(A)

**Figure:**
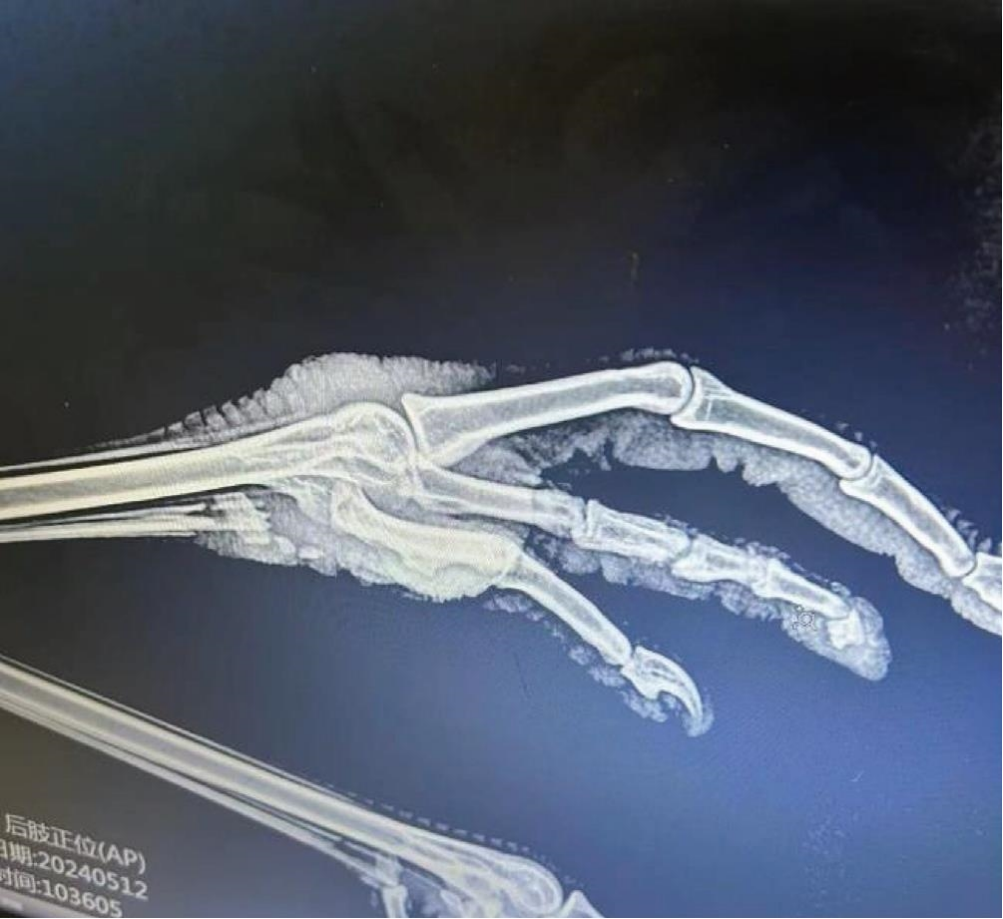
(B)

**Figure:**
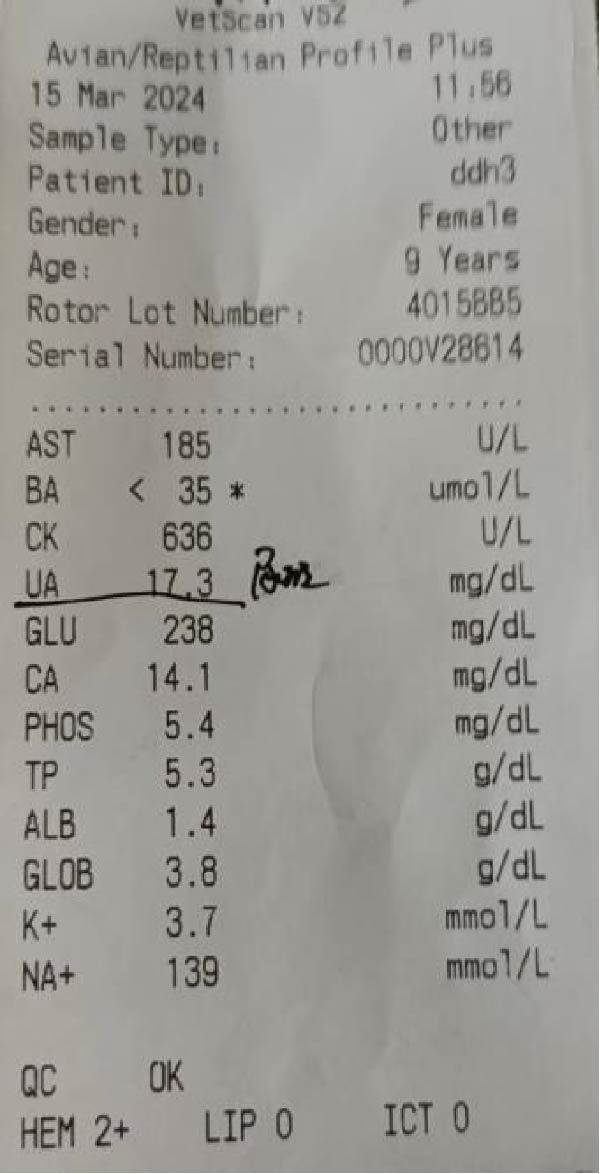
(C)

### 3.2. Metagenomics Sequencing of the Mixed Fecal Sample

To evaluate the gut microbes potentially associated with GA in red‐crowned cranes, metagenomics sequencing was performed on both RNA and DNA samples extracted from the mixed supernatant of four fecal samples. A total of 69.69 million and 102.19 million 150 bp clean reads were generated by RNA and DNA metagenomics sequencing, respectively (Supporting Information Table S2). Quality tests showed that the Q20 and Q30 rates were 98.78% and 96.12% for RNA data and 98.27% and 94.55% for DNA data. These results indicated a satisfactory quality of metagenomics sequencing.

Within 34.85 million paired‐end clean reads generated from the RNA sample, after removal of rRNA, host, and bacteria reads, only 9.64 million (27.66%), 9.41 million (27.01%), 0.66 million (1.89%) reads were left for RNA viruses (Supporting Information Table S3). Only *Myoviridae* (100%) was annotated at viral family level, while Enterobacteria phage phiEcoM‐GJ1 (93.22%) and Wuhan coneheads virus 1 (6.78%) were identified at species level (Figure 2A). Meanwhile, within 51.10 million paired‐end clean reads generated from the DNA sample, after removal of rRNA, host, and bacteria reads, 50.88 million (99.58%), 49.55 million (96.97%), 12.72 million (24.89%) reads were left for DNA viruses (Supporting Information Table S3). *Myoviridae* (84.21%), *Poxviridae* (9.15%), and *Retroviridae* (6.64%) were annotated at family level, while Enterobacteria phage phiEcoM‐GJ1 (83.03%), BeAn 58058 virus (9.02%), Human endogenous retrovirus K (6.54%), and pandoravirus salinus (1.40%) were detected at species level (Figure 2B). Remarkably, Enterobacteria phage phiEcoM‐GJ1 was detected as the predominant virus in both RNA and DNA samples extracted from red‐crowned crane feces.

Figure 2Metagenomics sequencing of feces from these red‐crowned cranes. (A,B) Detection of viruses by metagenomics sequencing using both RNA and DNA samples identified that Enterobacteria phage phiEcoM‐GJ1 is predominant in the feces of red‐crowned cranes. (C,D) Bacteria detection in the RNA and DNA samples. *Escherichia coli* and *Aeromonas hydrophila* are the most abundant bacteria. (E,F) Real‐time PCR amplification of top 5 bacteria detected in RNA and DNA samples by metagenomics sequencing as previously described [13]. The real‐time PCR results are largely consistent with metagenomics sequencing results.

**Figure:**
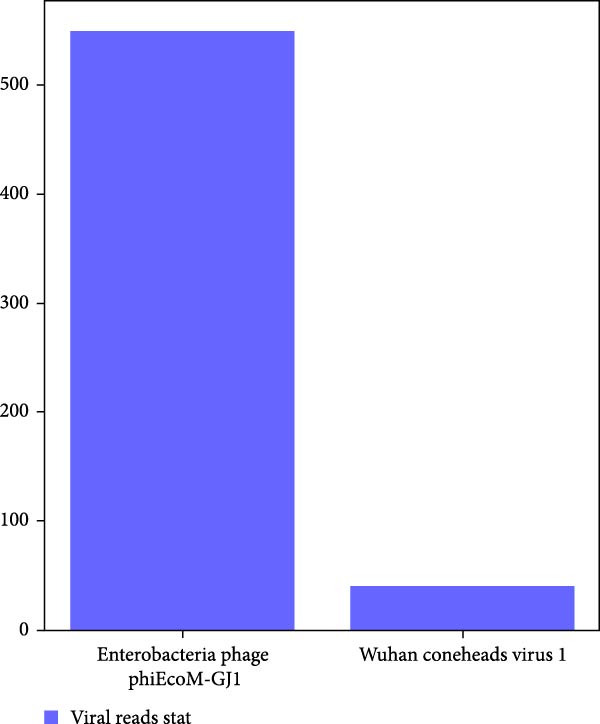
(A)

**Figure:**
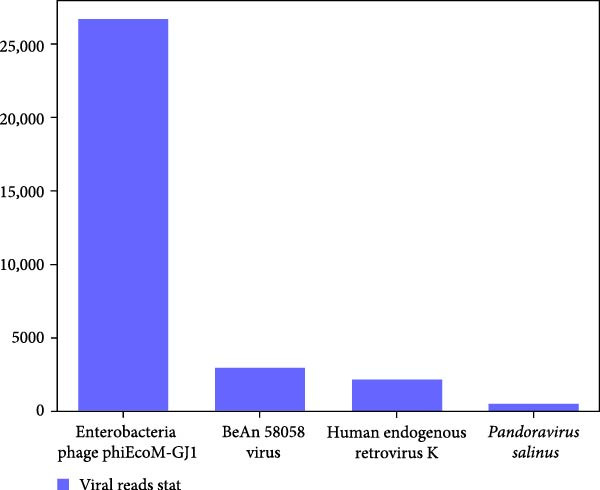
(B)

**Figure:**
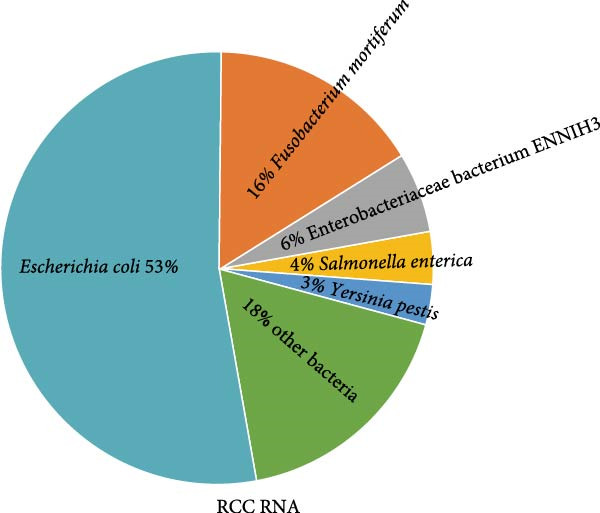
(C)

**Figure:**
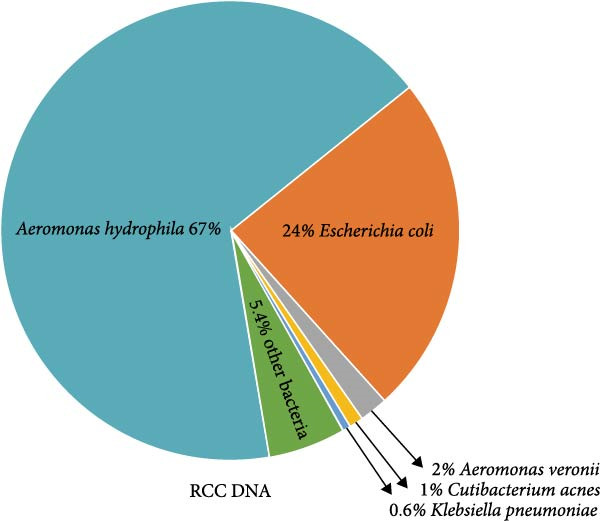
(D)

**Figure:**
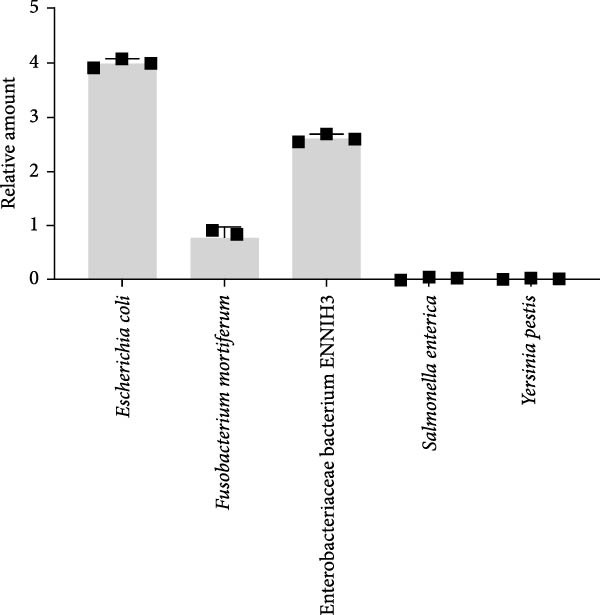
(E)

**Figure:**
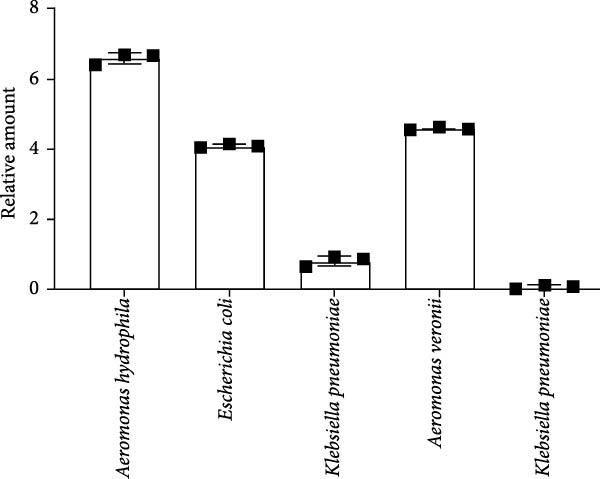
(F)

Noticeably, 8.75 million (25.12%) and 36. 83 million (72.08%) reads from RNA and DNA samples were bacteria. Considering that the Enterobacteria phage was the predominant virus, therefore, we also evaluated the obtained gut bacteria sequences. The results showed that *Escherichia coli* is predominant (53%) in RNA sample (Figure 2C), while *Aeromonas hydrophilia* (67%) and *Escherichia coli* (24%) are dominant in DNA sample (Figure 2D). These results were further confirmed by qPCR detections (Figure 2E,F). Noticeably, *Escherichia coli* was commonly detected in the DNA sample of each red‐crowned crane while *Aeromonas hydrophilia* was mainly detected in the RCC‐26 red‐crowned crane (Supporting Information Figure S1). Overall, the metagenomics sequening results suggested that GA in these red‐crowned cranes is unlikely associated with novel virus infection, but probably associated with gut microbiota dysbiosis, especially the imbalance of Enterobacteriaceae.

### 3.3. 16S rRNA Gene Sequencing of the Fecal *Samples*

To specifically evaluate the bacteria results of metagenomics sequencing, 16S rRNA gene sequencing was executed for one mixed and four individual (RCC‐26, RCC‐27, RCC‐30, RCC‐32) fecal supernatants. For these samples, 0.13 – 0.15 million clean reads (0.03 G – 0.04 G clean bases) were obtained (Supporting Information Table S4). The Q20 and Q30 rates were 97.02% – 97.75% and 92.01% – 93.64%, which suggested that the quality of the 16S rRNA gene sequencing was also satisfactory.

At the phylum level, firmicutes and proteobacteria were the predominant gut microbiota in these red‐crowned cranes, which accounted for 53.47% and 42.02% in the mixture fecal sample (Figure 3A). Similarly, they also dominanted the gut microbiota community in individual fecal samples (75.97% and 18.17% in the RCC‐26, 61.84% and 29.18% in RCC‐27, 9.80% and 90.13% in RCC‐30, 94.15% and 1.98% in RCC032) (Supporting Information Table S5). At the genus level, *Escherichia-Shigella* (40.48%), *Lactobacillus* (33.54%), *Enterococcus* (12.26%), *Staphylococcus* (3.70%), and Butyricococcaceae (1.50%) are the top 5 listed gut microorganisms in the mixture fecal sample (Figure 3B), which were confirmed by qPCR (Figure 3C). Similarly, *Escherichia-Shigella*, *Lactobacillus*, and *Enterococcus* were also detected as the most abundant gut flora in the fecal sample of each red‐crown crane (Table 1). At the species level, majority of them were unclassified by 16S rRNA sequencing (Supporting Information Figure S2). Overall, the 16S rRNA results are highly coincident with metagenomics results that these red‐crowned cranes suffered a disorder of gut microbiota.

Figure 3The relative abundances of bacteria in the feces of red‐crowned cranes detected by 16S rRNA gene sequencing. (A) The predominant bacterial phyla in the mixed fecal supernatant. (B) The predominant bacterial genera in the mixed fecal supernatant. (C) The relative levels of top 5 bacterial genera were also evaluated by real‐time PCR detections.

**Figure:**
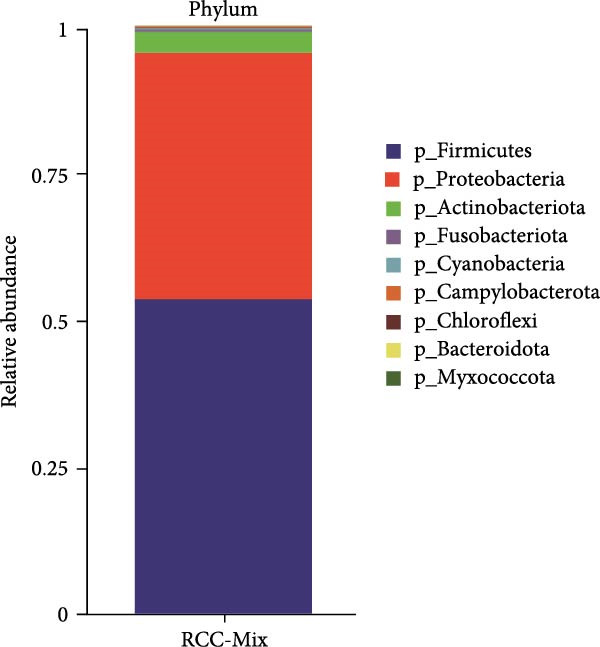
(A)

**Figure:**
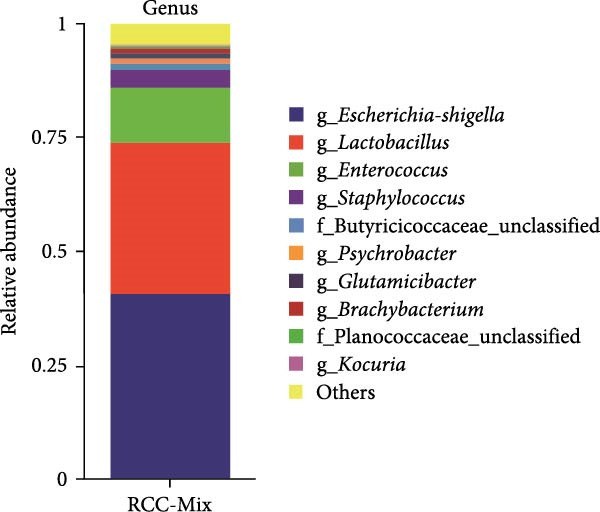
(B)

**Figure:**
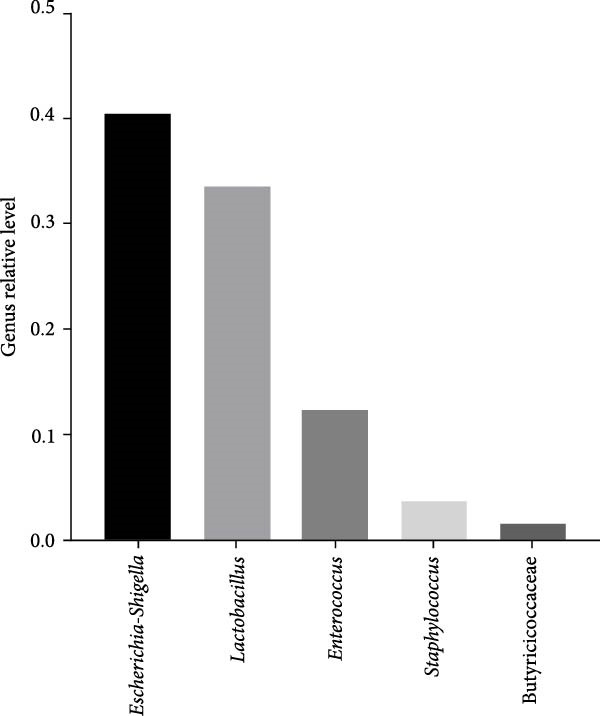
(C)

**Table 1:** The top 5 16S reads of gut microbiota at genus level.

Sample	Genus	Reads
RCC‐Mix	g*__Escherichia-Shigella*	23,387
	g*__Lactobacillus*	19,379
	g*__Enterococcus*	7084
	g*__Staphylococcus*	2139
	f__Butyricicoccaceae_unclassified	866

RCC‐26	g*__Paraclostridium*	20,503
	g*__Lactobacillus*	10,043
	g*__Aeromonas*	8639
	g*__Enterococcus*	5430
	g*__ZOR0006*	2616

RCC‐27	g*__Lactobacillus*	31,898
	g*__Escherichia-Shigella*	16,339
	g*__Fusobacterium*	2512
	g*__Brachybacterium*	730
	g_*_Clostridium_sensu_stricto_1*	716

RCC‐30	g*__Escherichia-Shigella*	52,019
	g*__Enterococcus*	5111
	g*__Staphylococcus*	465
	f__Enterobacteriaceae_unclassified	51
	g*__Bacillus*	39

RCC‐32	g*__Staphylococcus*	34,647
	g*__Enterococcus*	16,455
	g*__Lactobacillus*	2371
	g_*_Rothia*	1783
	g_*_Escherichia-Shigella*	1125

### 3.4. Bacteria Isolation and MALDI‐TOF/MS Identification

**Figure 4:**
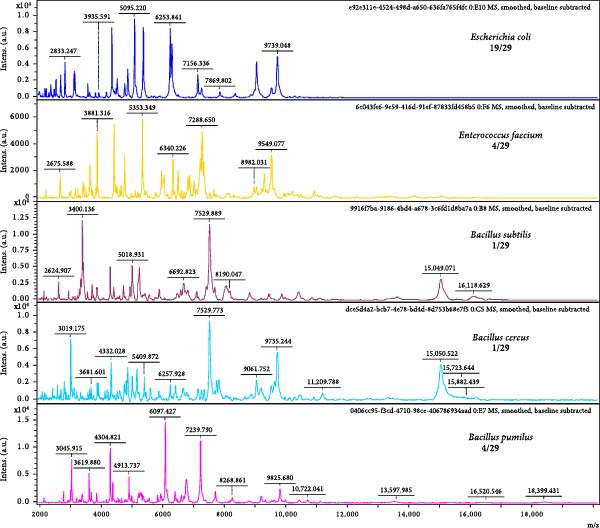
Identification of bacteria in the feces of red‐crowned cranes by MALDI‐TOF/MS. 29 bacteria were randomly selected and submitted to MALDI‐TOF/MS detection. *Esherichia coli* (19/29) was the most abundant bacteria, followed by *Enterococcus faecium* (4/29), *Bacillus pumilus* (4/29), *Bacillus subtilis* (1/29), and *Bacillus cereus* (1/29).

To further identify the exact bacteria in the mix fecal supernatant, bacteria were isolated using three culture plates and determined by MALDI‐TOF/MS. Five types of bacteria were identified in 29 randomly selected colonies using MALDI‐TOF/MS (Figure 4). There were 65.52% (19/29) colonies detected as *Escherichia coli*, including 7/12 (58.33%), 8/8 (100%) and 4/9 (44.44%) colonies from nutrient agar plate, Macconkey agar plate and blood agar plate, respectively (Table 2). Meanwhile, 13.79% (4/29), 13.79% (4/29), 3.45% (1/29), 3.45% (1/29) colonies were identified as *Enterococcus faecium*, *Bacillus pumilus*, *Bacillus subtilis*, *Bacillus cereus*, respectively (Table 2). These MALDI‐TOF/MS identification results were also consistent with metagenomics sequencing and 16S rRNA sequencing results.

**Table 2:** Bacteria identification by MALDI biotyper in mixed fecal sample with preexisting database.

No.	MALDI‐TOF results (best match)	Score values	Culture medium	Matched pattern	Metagenomics sequencing		16S rRNA sequencing
					DNA	RNA	
1	*Escherichia coli*	2.01	LB	*Escherichia coli DH5α BRL*	Top2 (24%)	Top1 (53%)	Top1 (40.5%)
2	*Escherichia coli*	1.94	LB	*Escherichia coli DH5α BRL*			
3	*Escherichia coli*	2.15	LB	*Escherichia coli* DSM 1576 DSM			
4	*Escherichia coli*	2.33	LB	*Escherichia coli* DSM 1576 DSM			
5	*Escherichia coli*	2.27	LB	*Escherichia coli* DSM 1576 DSM			
6	*Escherichia coli*	2.34	LB	*Escherichia coli* DSM 1576 DSM			
7	*Escherichia coli*	2.17	LB	*Escherichia coli* MB11464 1CHB			
8	*Escherichia coli*	1.82	MAC	*Escherichia coli* DH5*α* BRL			
9	*Escherichia coli*	2.23	MAC	*Escherichia coli* DH5*α* BRL			
10	*Escherichia coli*	2.13	MAC	*Escherichia coli* DH5*α* BRL			
11	*Escherichia coli*	1.97	MAC	*Escherichia coli* RV412_A1_2010_06a LBK			
12	*Escherichia coli*	2.30	MAC	*Escherichia coli* DH5*α* BRL			
13	*Escherichia coli*	2.24	MAC	*Escherichia coli DH5α BRL*			
14	*Escherichia coli*	2.38	MAC	*Escherichia coli* DSM 1576 DSM			
15	*Escherichia coli*	2.32	MAC	*Escherichia coli* DH5*α* BRL			
16	*Escherichia coli*	1.86	BAP	*Escherichia coli* DSM 682 DSM			
17	*Escherichia coli*	2.22	BAP	*Escherichia coli* DSM 1576 DSM			
18	*Escherichia coli*	2.11	BAP	*Escherichia coli* DSM 682 DSM			
19	*Escherichia coli*	2.27	BAP	*Escherichia coli* DSM 1576 DSM			

20	*Bacillus pumilus*	1.82	LB	*Bacillus pumilus* DSM 103904 DSM	0.00005%	—	Top13 (0.26%)
21	*Bacillus pumilus*	1.71	LB	*Bacillus pumilus* DSM 1794 DSM			
22	*Bacillus pumilus*	1.71	LB	*Bacillus pumilus* DSM 103904 DSM			
23	*Bacillus pumilus*	2.01	LB	*Bacillus pumilus* DSM 103904 DSM			

24	*Bacillus cereus*	1.81	LB	*Bacillus cereus thuringiensis* PG IV 17178035 1 MVD	0.006%	0.003%	Top13 (0.26%)

25	*Enterococcus faecium*	2.33	BAP	*Enterococcus faecium* 11037 CHB	0.03%	0.03%	Top3 (12.3%)
26	*Enterococcus faecium*	2.30	BAP	*Enterococcus faecium* 11037 CHB			
27	*Enterococcus faecium*	2.39	BAP	*Enterococcus faecium* 20218_1 CHB			
28	*Enterococcus faecium*	2.37	BAP	*Enterococcus faecium* 11037 CHB			

29	*Bacillus subtilis*	1.87	BAP	*Bacillus subtilis* DSM 5611 DSM	0.004%	0.005%	Top13 (0.26%)

Abbreviations: BAP, blood agar plate; LB, nutrient agar plate (Luria–Bertani medium); MAC, Macconkey agar plate; —, not found.

### 3.5. Treatment

Therapeutic diagnosis of gut microbiota dysbiosis were adopted by the Animal hospital of Yangzhou University. The anti‐uricacid and antibacteria treatments using plantain herb, doxycycline, Vitamin AD3, and multivitamin B led to a full behavioral recovery within 1 month (Supporting Information Figure S3). Overall, this case study provides the first clue that gut microbiota dysbiosis is closely associated with GA in red‐crowned cranes.

## 4. Discussion

Health issue is a major cause for the continuous decreasing number of red‐crowned cranes. GA is detrimental to human and animal health, which could be regulated by gut microbiota [9]. Noticeably, a latest study revealed that gut microbial metabolites influence osteoarthritis progression and intestinal farnesoid X receptor is a viable therapeutic target to ameliorate osteoarthritis by increasing intestinal L cell‐secreted glucagon‐like peptide 1, which uncovered the existence of a functional and targetable gut‐joint axis in human [12]. In this study, we firstly reported a GA case in red‐crowned cranes from the Wildlife Rescue Center of Suining County of Jiangsu Province. In addition, metagenomics sequencing and 16S rRNA sequencing were utilized to explore the gut microbiota in fecal samples from these red‐crowned cranes. High throughput sequencing results indicated that GA in these red‐crowned cranes is probably correlated with gut microbiota dysbiosis, which was further supported by bacteria isolation and MALDI‐TOF/MS identification. Moreover, anti‐uricacid and antibacteria treatments were executed leading to a full behavioral recovery of GA. Even though gut microbiota dysbiosis has been proved to be implicated in osteoarthritis in human, the findings from this study provide first clue on the gut‐joint axis in red‐crowned cranes supporting that gut microbiota dysbiosis is implicated in GA of red‐crowned cranes.

GA is an autoinflammatory arthropathy with hyperuricemia as the prerequisite for disease development [19]. In human, GA manifests as an acute episode of painful arthritis due to deposition of MSU crystals in the joints, especially the big toe [20]. Specific‐pathogen‐free C57BL/6 mice may be used as animal model to study MSU induced acute GA, which caused mechanical, thermal hyperalgesia, and paw swelling in mice [7]. Chicken serves as a better model than mice to study GA because both human and poultry lack of uricase and have high incidence of GA [8]. MSU‐injection into the ankle joint of chicken could induce spontaneous pain behaviors, including standing on one leg, limping, and sitting. Edema and mechanical pain hypersensitivity also occurred in the chicken with GA [8]. However, there was no GA study in red‐crowned crane. Here we reported for the first time the occurrence of GA in red‐crowned cranes from a Wildlife Rescue Center in Suining County of Jiangsu Province, which accompanied with limping, ankle swelling and hyperuricemia.

High‐throughput sequencing is independent of culture and can massively foster the discovery of novel pathogens comparing to traditional methods. Metagenomics sequencing not only helps to identify potential zoonotic and emerging pathogens, but also enables to identify the etiology of diseased red‐crowned cranes [2, 4]. For instance, viral metagenomics sequencing of feces from wild and captive red‐crowned cranes identified vertebrate viruses including *Picornaviridae*, *Parvoviridae*, *Circoviridae*, and *Caliciviridae*. Simultaneously, insect, crustacean shellfish and plant viruses, including *Dicistroviridae*, *Iridoviridae*, *Bidnaviridae*, *Phycodnaviridae*, *Geminiviridae*, *Nanoviridae*, *Tombusviridae*, were also detected [4]. Another virome characterization identified DNA (*Genomoviridae* and *Parvoviridae*) and RNA (*Picornaviridae*) viruses in the feces of red‐crowned cranes that were possibly the causative agents for diarrhea in red‐crowned crane [2]. Noticeably, a recent study showed that a novel goose astrovirus is associated with acute gosling gout disease [21]. In this study, even though *Aeromonas hydrophilia* was also detected as a major bacterium in the mixed fecal sample by metagenomics sequencing (Figure 2D,F), it’s only dominant in one red‐crowned crane (Supporting Information Figure S1 and Table 1). In contrast, *Escherichia coli* (> 20%) and Enterobacteria phage phiEcoM‐GJ1 (> 83%) were the extensively detected bacteria and virus, which were obviously higher than normal levels [10, 22]. Noticeably, the phiEcoM‐GJ1 is a lytic phage that attacks *Esherichia coli* [23]. Therefore, our findings supported that the imbalance of gut microbiota is probably associated with GA in red‐crowned cranes.

Several studies have provided treatment regimens for GA. A 6‐week doxycycline and moxifloxacin regimen could significantly improve joint disability in human [24]. The calcium‐stimulated adenylyl cyclase subtype I (ACI) inhibitor NB001 could reduce both the spontaneous pain behaviors and the mechanical pain hypersensitivity in Chicken [8]. Gentiopicroside could be used to treat acute GA in mice based on its anti‐inflammatory and analgesic properties [7]. In addition, vitamins (such as D3) played protective roles in acute inflammation and were relevant to GA [6, 25]. The n‐butanol extracted from *Calendula stellate* (*Asteraceae* family) growing in Northeast Algeria also possesses antiarthritic activity [26]. Besides that, Chinese herbal medicines, especially phenolic, flavonoid, terpenoid and alkaloid compounds, were also considered as key ingredients to improve acute GA [27]. In this study, we applied a combined treatment of Chinese and Western medicines (plantain herb, doxycycline and vitamins) to cure GA in these red‐crowned cranes, leading to a satisfactory outcome. Therefore, this study also provides an effective therapeutic regimen for GA in red‐crowned cranes.

This study still has several limitations. At first, even though several potential correlated pathogens were identified and isolated in this study, it’s not possible to evaluate the direct causes of these pathogens for GA in red‐crowned crane via animal challenge studies because the red‐crowned crane is the National Class I Key Wildlife Protection of China. Second, all the four red‐crowned cranes from the Wildlife Rescue Center of Suining County of Jiangsu Province suffered from GA at meantime, therefore, no negative control could be set up when we performed the high‐throughput sequencings. Third, even though metagenomics sequencing and 16S rRNA gene sequencing help to screen potentially associated pathogens, due to technique limits and no enough replicates, the high‐throughput sequencing results might not exact reflect the real infection status. Fourth, after a full behavioral recovery of these red‐crowned cranes, we did not get the permission to collect more samples to re‐evaluate the gut microbiota in these red‐crowned cranes. Fifth, gut microbiota dysbiosis could also be influenced by environment, living modes (captive and free) or dietary factors (such as antibiotic exposure), which were not determined in this study. Therefore, the direct correlation between gut microbiota and GA in red‐crowned cranes still requires comprehensive investigations.

In summary, this is the first study to report GA in red‐crowned cranes. Metagenomics sequencing and 16S rRNA gene sequencing were applied to evaluate the gut microbiota in these red‐crowned cranes, which were further confirmed by MALDI‐TOF/MS identification. Our findings provided first clue on the gut‐joint axis in red‐crowned cranes supporting that gut microbiota dysbiosis is closely associated with GA in red‐crowned cranes.

## Disclosure

All authors read and approved the final version of the manuscript.

## Conflicts of Interest

The authors declare no conflicts of interest.

## Author Contributions

Wenbin Bao and Nanhua Chen conceived, designed, and supervised this study. Hong Lin and Xiaoyang Zhu performed sample collection and experimental studies. Hong Lin, Xiaoyang Zhu, and Nanhua Chen participated in data analyses. Wenbin Bao, Nanhua Chen, and Jianzhong Zhu contributed reagents/materials/analysis. Wenbin Bao, Nanhua Chen, and Jianzhong Zhu provided fundings for this study. Hong Lin and Nanhua Chen wrote the first version of the manuscript. Hong Lin and Xiaoyang Zhu contributed equally to this manuscript.

## Funding

This study was supported by National Key R&D Program of China (Grant 2023YFD1800504), Higher Education Institutions Basic Science (Natural Science) Research General Program of Jiangsu Province (Grant 24KJB230005), Priority Academic Program Development of Jiangsu Higher Education Institutions (PAPD), and 111 Project D18007.

## Supporting Information

Additional supporting information can be found online in the Supporting Information section.

## Supporting information


**Supporting Information 1** Table S1. The primers used for bacteria detection in this study.

**Supporting Information 2** Table S2. Metagenomics sequencing quality.

**Supporting Information 3** Table S3. Metagenomic sequencing data filtration.

**Supporting Information 4** Figure S1. Relative amounts of *Escherichia coli* and *Aeromonas hydrophila* in DNA sample from each red‐crowned crane detected by qPCR. (A) Relative amounts of *Escherichia coli*. (B) Relative amounts of *Aeromonas hydrophila*.

**Supporting Information 5** Table S4. 16S rRNA gene sequencing quality.

**Supporting Information 6** Table S5. The 16S reads of gut microbiota at phylum level.

**Supporting Information 7** Figure S2. The relative abundances of bacteria in the feces of red‐crowned cranes detected by 16S rRNA gene sequencing. The predominant bacterial species in the mixed fecal supernatant.

**Supporting Information 8** Figure S3. Clinical observation of a representative red‐crowned crane before and after therapeutic regimen. Limping was observed in the red‐crowned crane before treatment but not after treatment.

## Data Availability

The metagenomics sequencing and 16S rRNA gene sequencing data in this study can be accessed in the SRA database with accession No. of PRJNA1313004 and PRJNA1311824 (https://submit.ncbi.nlm.nih.gov/subs/sra/). The data underlying this article are available in this article and its supporting information.
