# Supplementary material for: High‐Throughput Sequencings Revealed That Gut Microbiota Dysbiosis is Implicated in Gouty Arthritis of Red‐Crowned Crane (Grus japonensis)
Source: Transbound Emerg Dis. 2025 Dec 15;2025:2422900. doi: 10.1155/tbed/2422900 (PMC12703207; doi:10.1155/tbed/2422900)
Supplement: Supplementary file 1 — Supporting Information 1 Table S1. The primers used for bacteria detection in this study. [file TBED-2025-2422900-s007.docx]

Table S1. The primers used for bacteria detection in this study.

| Name | Sequence of the primer | Product size (bp) |
| --- | --- | --- |
| *Escherichia coli* | F: 5'-GTTAATACCTTTGCTCATTGA-3'  R: 5'-ACCAGGGTATCTAATCCTGTT-3' | 130 |
| *Turicibacter sp. H121* | F: 5'- CAGACGGGGACAACGATTGGA-3'  R: 5'-TACGCATCGTCGCCTTGGTA-3' | 182 |
| *Salmonella enterica* | F: 5'-GCTTTCTCTACTTAAC-3'  R: 5'-GTAATGGAATGACGAACAT -3' | 180 |
| *Yersinia pestis* | F: 5'-AAGACTGGGGGCTAACGAAT-3'  R: 5'-CCAGAAAACCAAACATGC-3' | 200 |
| *Bacillus anthracis* | F: 5'-GAAGCTTGGTTAGAGTAT-3'  R: 5'-CATCTGGATCCCCTACTTTGTATAGT-3' | 162 |
| *Enterobacteriaceae* | F: 5'-GTGCCAGCAGCCGCGGTAA-3'  R: 5'-GCCTCAAGGGCACAACCTCCAAG-3' | 119 |
| *Clostridium butyricum* | F: 5'-AGCAACGCGAAGAACCTTAC-3'  R: 5'-ATTTGACGTCATCCCCACCT-3' | 188 |
| *Fusobacterium mortiferum* | F: 5'-AAGAGACCAAACCAGCCTCA-3'  R: 5'-CTCAGCCACCCTTCTTAGCT-3' | 125 |
| *Aeromonas hydrophila* | F: 5'-CGCTCCAAGATCCCGGTGAA-3'  R: 5'-AGGAAGCCGCTCAGGGTCAG -3' | 125 |
| *Cutibacterium acnes* | F: 5'-"ATGAAAAACATTCGTTTGAT "-3'  R: 5'-TCGTTGTCCTTGCGATACCT-3' | 120 |
| *Aeromonas vickerii* | F: 5'-ATGGCGAAACGCATTGATCG-3'  R: 5'-TCGTTGTCCTTGCGATACCT-3' | 120 |
| *Klebsiella pneumoniae* | F: 5'-ACTGGAACGTAAAAGGTA-3'  R: 5'-GCGAAATCGAAGTAGATC-3' | 150 |
| *Burkholderia pseudomallei* | F: 5'-TCGACGTTCGACGGCCGCT-3'  R: 5'-TGAGATCGCCCTTCGCGTACGC-3' | 188 |
| *Enterococcus casseliflavus* | F: 5'-GGAGCTTGCTCCACCGAA-3'  R: 5'-TTTCTTCCATGCGGAAAATAGT-3' | 192 |
