# Supplementary material for: High‐Throughput Sequencings Revealed That Gut Microbiota Dysbiosis is Implicated in Gouty Arthritis of Red‐Crowned Crane (Grus japonensis)
Source: Transbound Emerg Dis. 2025 Dec 15;2025:2422900. doi: 10.1155/tbed/2422900 (PMC12703207; doi:10.1155/tbed/2422900)
Supplement: Supplementary file 2 — Supporting Information 2 Table S2. Metagenomics sequencing quality. [file TBED-2025-2422900-s001.docx]

Table S2. Metagenomics sequencing quality.

| QC | RCC-RNA mixture sample | | RCC-DNA mixture sample | |
| --- | --- | --- | --- | --- |
|  | Before filtering | After filtering | Before filtering | After filtering |
| Total_reads | 82490756 | 69692272 | 107617458 | 102193230 |
| Total_bases | 11.52 G | 5.78 G | 15.03 G | 11.18 G |
| Q20_bases | 10.26 G | 5.71 G | 14.16 G | 10.99 G |
| Q30_bases | 9.03 G | 5.55 G | 13.47 G | 10.9 G |
| Q20_rate | 89.04% | 98.78% | 94.17% | 98.27% |
| Q30_rate | 78.32% | 96.12% | 87.31% | 94.55% |
