# Supplementary material for: High‐Throughput Sequencings Revealed That Gut Microbiota Dysbiosis is Implicated in Gouty Arthritis of Red‐Crowned Crane (Grus japonensis)
Source: Transbound Emerg Dis. 2025 Dec 15;2025:2422900. doi: 10.1155/tbed/2422900 (PMC12703207; doi:10.1155/tbed/2422900)
Supplement: Supplementary file 3 — Supporting Information 3 Table S3. Metagenomic sequencing data filtration. [file TBED-2025-2422900-s002.docx]

Table S3. Metagenomic sequencing data filtration.

| Sample | Clean reads | rm_rRNA | rm_host | rm_bacteria |
| --- | --- | --- | --- | --- |
| RCC-RNA | 34846136(100%) | 9637322(27.66%) | 9411919(27.01%) | 657472(1.89%) |
| RCC-DNA | 51096615(100%) | 50882102(99.58%) | 49550460(96.97%) | 12718254(24.89%) |
