# Supplementary material for: High‐Throughput Sequencings Revealed That Gut Microbiota Dysbiosis is Implicated in Gouty Arthritis of Red‐Crowned Crane (Grus japonensis)
Source: Transbound Emerg Dis. 2025 Dec 15;2025:2422900. doi: 10.1155/tbed/2422900 (PMC12703207; doi:10.1155/tbed/2422900)
Supplement: Supplementary file 4 — Supporting Information 4 Figure S1. Relative amounts of Escherichia coli and Aeromonas hydrophila in DNA sample from each red‐crowned crane detected by qPCR. (A) Relative amounts of Escherichia coli. (B) Relative amounts of Aeromonas hydrophila. [file TBED-2025-2422900-s008.pdf]

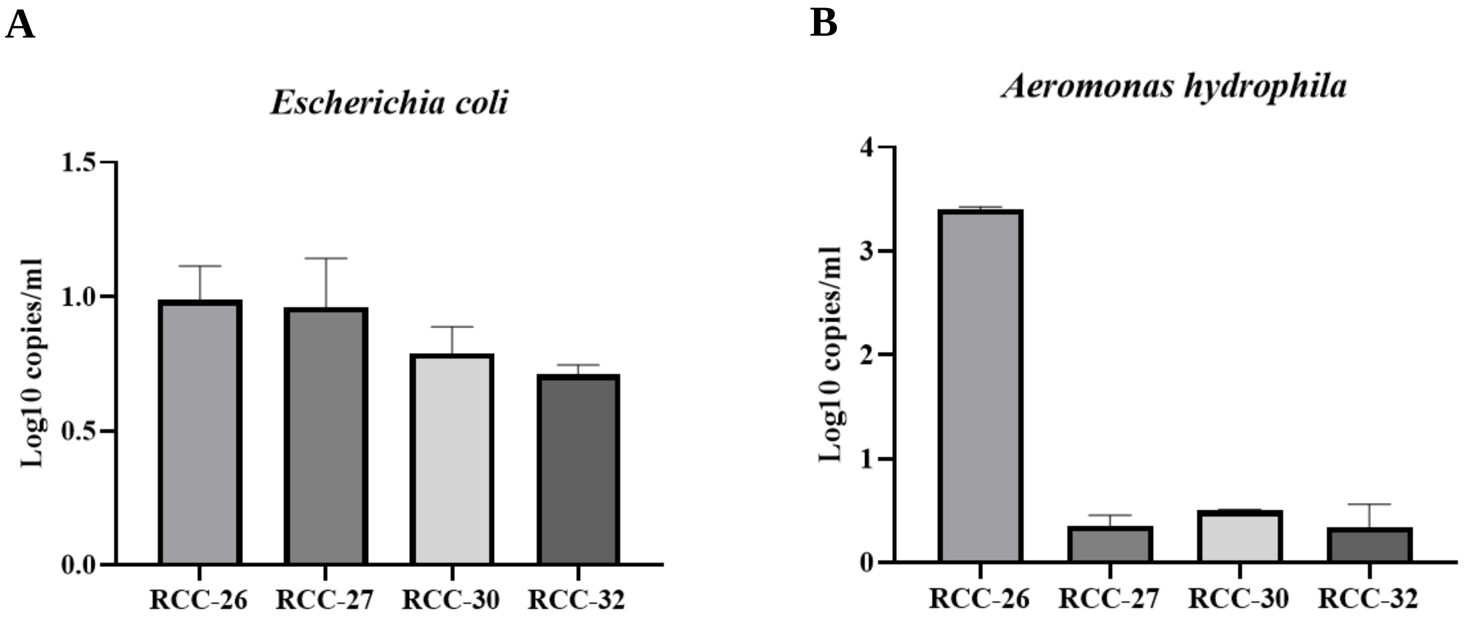

Figure S1. Relative amounts of *Escherichia coli* and *Aeromonas hydrophila* in each DNA sample extracted from red-crowned cranes detected by qPCR. (A) Relative amounts of *Escherichia coli*. (B) Relative amounts of *Aeromonas hydrophila*.
