# Supplementary material for: High‐Throughput Sequencings Revealed That Gut Microbiota Dysbiosis is Implicated in Gouty Arthritis of Red‐Crowned Crane (Grus japonensis)
Source: Transbound Emerg Dis. 2025 Dec 15;2025:2422900. doi: 10.1155/tbed/2422900 (PMC12703207; doi:10.1155/tbed/2422900)
Supplement: Supplementary file 5 — Supporting Information 5 Table S4. 16S rRNA gene sequencing quality. [file TBED-2025-2422900-s005.docx]

Table S4. 16S rRNA gene sequencing quality

| Sample | Raw_reads | Raw_bases | Clean_reads | Clean_bases | Q20 | Q30 |
| --- | --- | --- | --- | --- | --- | --- |
| RCC-Mix | 157966 | 0.04 G | 151518 | 0.04 G | 97.24% | 92.56% |
| RCC-26 | 137560 | 0.03 G | 132070 | 0.03 G | 97.31% | 92.67% |
| RCC-27 | 156462 | 0.04 G | 149972 | 0.04 G | 97.02% | 92.01% |
| RCC-30 | 159186 | 0.04 G | 152012 | 0.04 G | 97.75% | 93.64% |
| RCC-32 | 138362 | 0.03 G | 131732 | 0.03 G | 97.29% | 92.75% |
