# Supplementary material for: High‐Throughput Sequencings Revealed That Gut Microbiota Dysbiosis is Implicated in Gouty Arthritis of Red‐Crowned Crane (Grus japonensis)
Source: Transbound Emerg Dis. 2025 Dec 15;2025:2422900. doi: 10.1155/tbed/2422900 (PMC12703207; doi:10.1155/tbed/2422900)
Supplement: Supplementary file 6 — Supporting Information 6 Table S5. The 16S reads of gut microbiota at phylum level. [file TBED-2025-2422900-s006.docx]

Table S5. The 16S reads of gut microbiota at phylum level.

| Phylum | RCC-M | RCC-26 | RCC-27 | RCC-30 | RCC-32 |
| --- | --- | --- | --- | --- | --- |
| p__Firmicutes | 30892 | 43893 | 35726 | 5663 | 54396 |
| p__Proteobacteria | 24274 | 10500 | 16860 | 52070 | 1145 |
| p__Actinobacteriota | 2107 | 1845 | 2198 | 33 | 1947 |
| p__Fusobacteriota | 190 | 716 | 2603 | 0 | 0 |
| p__Cyanobacteria | 134 | 672 | 57 | 8 | 256 |
| p__Campilobacterota | 113 | 55 | 189 | 0 | 0 |
| p__Chloroflexi | 38 | 29 | 101 | 0 | 30 |
| p__Bacteroidota | 14 | 26 | 31 | 0 | 0 |
| p__Myxococcota | 12 | 0 | 0 | 0 | 0 |
| p__Deinococcota | 0 | 0 | 9 | 0 | 0 |
| p__Planctomycetota | 0 | 38 | 0 | 0 | 0 |
