# Supplementary material for: High‐Throughput Sequencings Revealed That Gut Microbiota Dysbiosis is Implicated in Gouty Arthritis of Red‐Crowned Crane (Grus japonensis)
Source: Transbound Emerg Dis. 2025 Dec 15;2025:2422900. doi: 10.1155/tbed/2422900 (PMC12703207; doi:10.1155/tbed/2422900)
Supplement: Supplementary file 7 — Supporting Information 7 Figure S2. The relative abundances of bacteria in the feces of red‐crowned cranes detected by 16S rRNA gene sequencing. The predominant bacterial species in the mixed fecal supernatant. [file TBED-2025-2422900-s003.pdf]

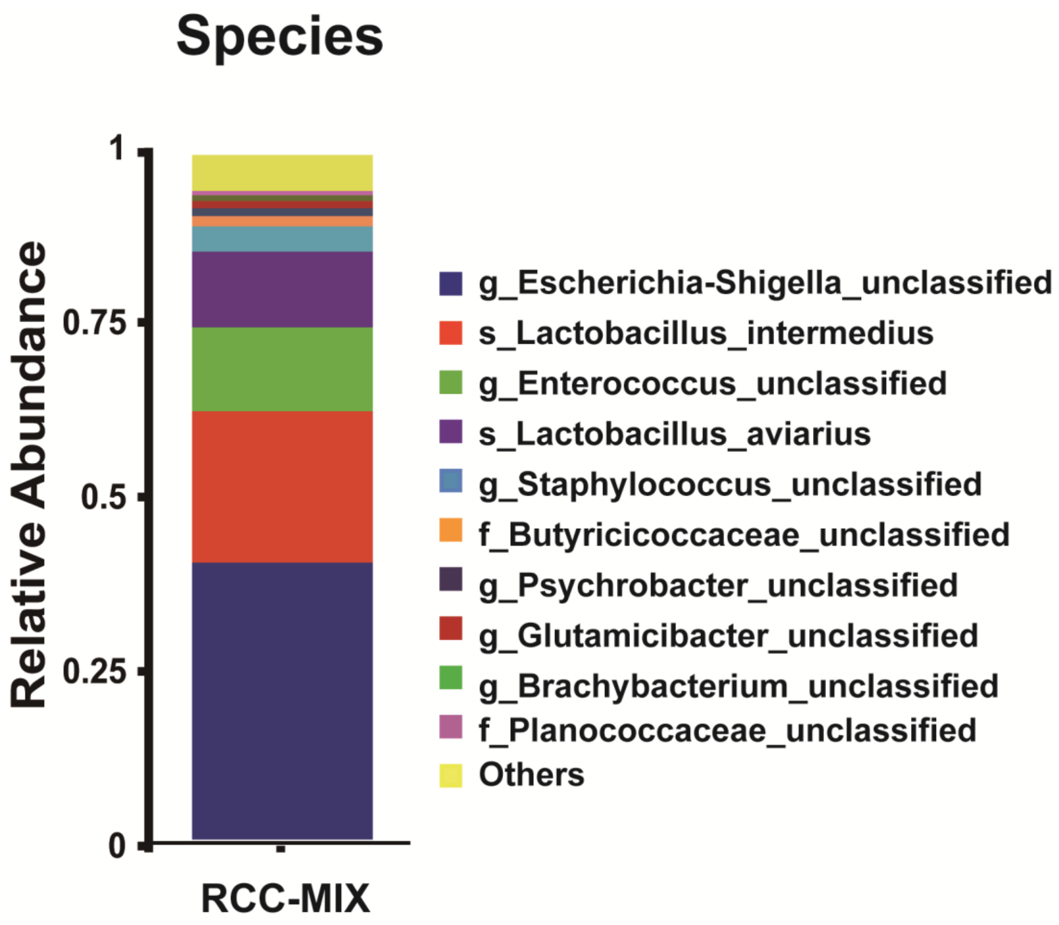

Figure S2. The relative abundances of bacteria in the feces of red-crowned cranes detected by 16S rRNA gene sequencing. The predominant bacterial species in the mixed fecal supernatant.
