# Supplementary material for: High‐Throughput Sequencings Revealed That Gut Microbiota Dysbiosis is Implicated in Gouty Arthritis of Red‐Crowned Crane (Grus japonensis)
Source: Transbound Emerg Dis. 2025 Dec 15;2025:2422900. doi: 10.1155/tbed/2422900 (PMC12703207; doi:10.1155/tbed/2422900)
Supplement: Supplementary file 8 — Supporting Information 8 Figure S3. Clinical observation of a representative red‐crowned crane before and after therapeutic regimen. Limping was observed in the red‐crowned crane before treatment but not after treatment. [file TBED-2025-2422900-s004.pdf]

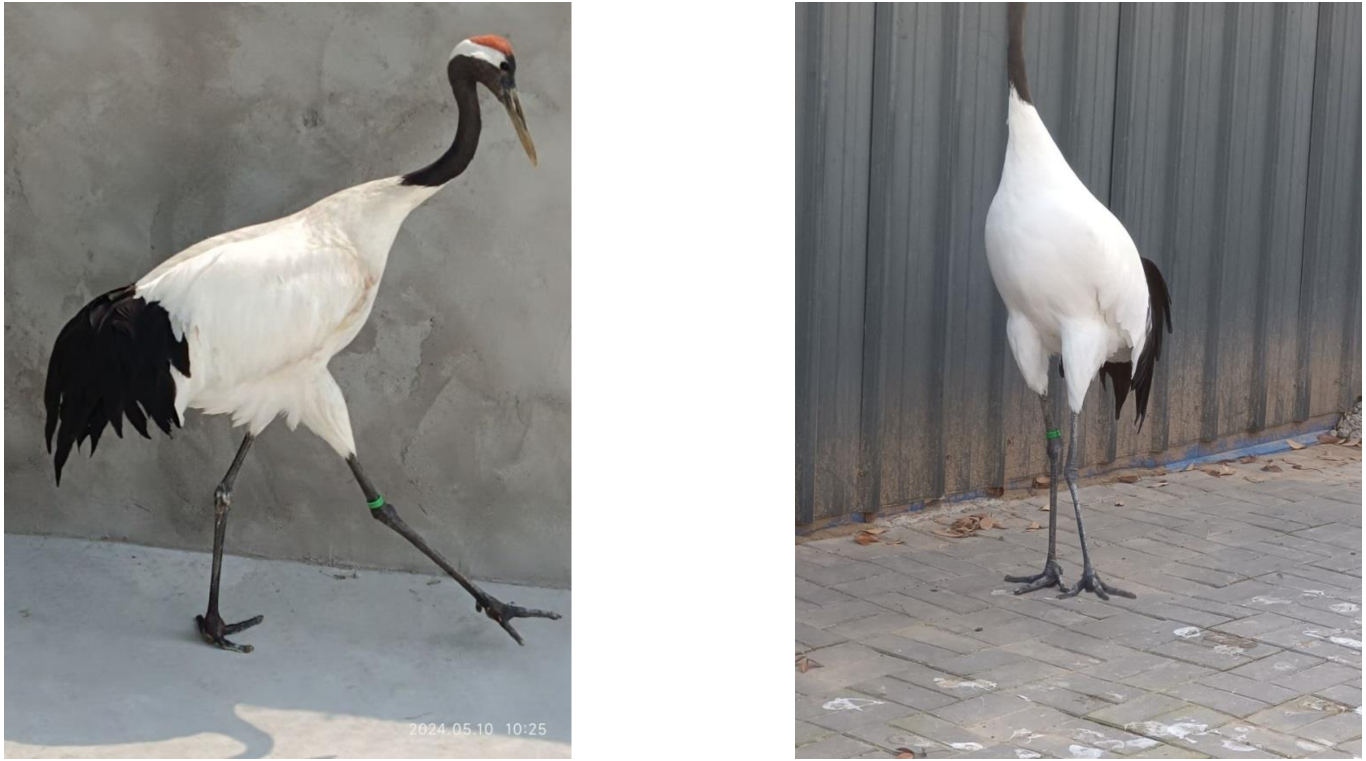

Before treatment After treatment

Figure S3. Clinical observation of a representative red-crowned crane before and after therapeutic regimen. Limping was observed in the red-crowned crane before treatment but not after treatment.
